# Understanding and engineering beneficial plant–microbe interactions: plant growth promotion in energy crops

**DOI:** 10.1111/pbi.12279

**Published:** 2014-11-28

**Authors:** Kerrie Farrar, David Bryant, Naomi Cope-Selby

**Affiliations:** Institute of Biological, Environmental & Rural Sciences (IBERS), Aberystwyth UniversityAberystwyth, UK

**Keywords:** bacterial endophyte, sustainable agriculture, biofertilization, symbiosis, plant–microbe signalling, LEANOME

## Abstract

Plant production systems globally must be optimized to produce stable high yields from limited land under changing and variable climates. Demands for food, animal feed, and feedstocks for bioenergy and biorefining applications, are increasing with population growth, urbanization and affluence. Low-input, sustainable, alternatives to petrochemical-derived fertilizers and pesticides are required to reduce input costs and maintain or increase yields, with potential biological solutions having an important role to play. In contrast to crops that have been bred for food, many bioenergy crops are largely undomesticated, and so there is an opportunity to harness beneficial plant–microbe relationships which may have been inadvertently lost through intensive crop breeding. Plant–microbe interactions span a wide range of relationships in which one or both of the organisms may have a beneficial, neutral or negative effect on the other partner. A relatively small number of beneficial plant–microbe interactions are well understood and already exploited; however, others remain understudied and represent an untapped reservoir for optimizing plant production. There may be near-term applications for bacterial strains as microbial biopesticides and biofertilizers to increase biomass yield from energy crops grown on land unsuitable for food production. Longer term aims involve the design of synthetic genetic circuits within and between the host and microbes to optimize plant production. A highly exciting prospect is that endosymbionts comprise a unique resource of reduced complexity microbial genomes with adaptive traits of great interest for a wide variety of applications.

## Introduction

To meet demand for sustainable alternatives to fossil fuels, dedicated energy crops that produce high annual biomass yields on low-quality land and without the need for fertilizer and pesticide inputs are being developed. Desirable energy crop traits include efficient low-cost establishment, rapid growth and high-biomass yields in the absence of chemical inputs, abiotic and biotic stress tolerance and perenniality (Lewandowski *et al.*, 2003). Primary candidates include the tall grasses, for example Miscanthus and switchgrass, and fast-growing trees such as Poplar and willow (Simmons *et al.*, 2008; Tuck *et al.*, 2006). In the near term, a number of crops domesticated for food and forage are also being grown specifically for bioenergy, notably sugar cane and Sorghum (Heaton *et al.*, 2008). In order to maximize net energy, outputs per unit of land novel approaches to boost biomass yields are required, including the manipulation of plant–microbe interactions.

Boosting crop yields has for several decades been the domain of the chemical industry. The green revolution, which arose during the 1940s and 1960s, included the development of nitrogen fertilizer derived from the Haber–Bosch process, phosphates and various other nutrients and pesticides (Tilman, 1998). Modern crops have largely been bred in conjunction with these economically and energetically costly chemicals, and therefore, have been selected to produce high yields in their presence, potentially at the expense of beneficial plant–microbe interactions hosted by their ancestors. Indeed, this may contribute to the reduction in competitive advantage many crops experience, to the point they are dependent on humans for their distribution. However, in an age of rapid population growth and climate change, alternative solutions are required to maintain and increase crop yields sustainably, without a concurrent increase in resource utilization (Tikhonovich and Provorov, 2011). These new approaches will require application of biological solutions, including the manipulation and exploitation of beneficial plant–microbe interactions. In contrast to crops that have been bred for food, the majority of dedicated perennial bioenergy crops are largely undomesticated and so there is an opportunity to harness relationships which may have been inadvertently lost through agronomic practice and intensive plant breeding (Finlay, 2008). To conserve the best land for food production, energy crops must be grown on marginal land, and must therefore tolerate a range of abiotic and biotic stresses (Jones *et al.*, 2014). Furthermore, energy crops are ideal for developing and evaluating novel technologies and applications as they are not consumed by humans. They will therefore provide important data about the safety of the use of bacteria to boost crop yields, which may then be applied more widely.

Overwhelmingly, research into plant–microbial interactions has focused on three categories of plant–microbe interactions: the ancient symbiosis between land plants and arbuscular mycorrhizae (AM, Smith and Smith, 2011), nitrogen fixation by rhizobia within the nodules of legume roots (Oldroyd *et al.*, 2011) and pathogenesis (Dodds and Rathjen, 2010; Kachroo and Robin, 2013; Wirthmueller *et al.*, 2013). These systems are now well characterized and provide insights into common and diverged signalling pathways involved in plant–microbe interactions. However, symbiosis is the norm rather than the exception, and so understanding plant–microbe interactions is of fundamental importance to gaining insights into plant evolution and adaptation (Hirsch, 2004). Plants are constantly interacting with a range of benign and parasitic organisms including bacteria, fungi and invertebrates in the soil. Complex relationships based on reciprocal signalling between diverse microbial consortia and plants abound both in the rhizosphere and within the plant itself (Badri *et al.*, 2009; Evangelisti *et al.*, 2014).

The spectrum of plant–microbe interactions is highly complex, comprising diverse microbial species, potentially acting as consortia (Hirsch, 2004). Apart from a few specialized examples, such as the legume–rhizobia interaction, monospecific interactions are considered to be the exception. These complex communities are very dynamic and may include opportunistic plant or human pathogens that are repressed under normal conditions (Berg *et al.*, 2005). Consortia may be governed by the presence of functional groups to maintain resilience rather than selection of specific microbial species and may involve tripartite interactions, for example between plant, fungi and bacteria (Bonfante and Anca, 2009; Dames and Ridsdale, 2012).

Developments in methodology are essential to this field of research. Historically only readily culturable species were studied, with different media and growth conditions required for different classes of microbes (Stewart, 2012; Vartoukian *et al.*, 2010). A number of endosymbionts such as mycorrhizae are not amenable to culture in isolation and must be grown in the presence of host tissue (Hildebrandt *et al.*, 2002). Methodological advances such as fluorescent tagging have been critical to the study of bacterial endophytes (Elbeltagy *et al.*, 2001), enabling clear visualization of small numbers of cells within the host, but remain out of reach for uncultured species. There is a growing interest in uncultured microbes, as these potentially represent ‘obligate endophytes’ which live their entire life cycle within the plant tissues. With the advent of next-generation sequencing, this fascinating group is gradually becoming accessible to study, and consequently, the body of data is accumulating (Bulgarelli *et al.*, 2012).

While plants and microbes have traditionally been studied and manipulated separately, understanding the interactions between the plant and its microbial symbionts requires a more holistic approach. Computational integration of different data types will be required to enable dissection of this complex and dynamic system. The aim of this review is firstly to summarize our current knowledge about the contribution of both plant and microbe to beneficial plant–microbe interactions in nonlegumes, and secondly to discuss opportunities and challenges ahead in the manipulation of plant–microbe interactions, in particular endophytic bacteria, to optimize production from biomass crops.

## Symbiosis—living together

**Figure 1:**
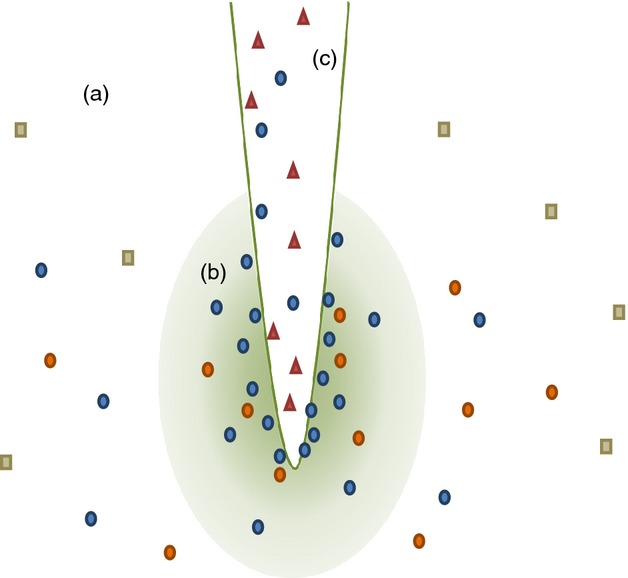
Plant–Microbe associations. Free-living bacteria in the soil (a), the rhizosphere population (b) and endosymbionts within the root (c) of a plant host may be a true continuum, with a subset of soil bacteria attracted to the rhizosphere (circles). A smaller number are able to enter the host and exist as endophytes (blue circles). The nature of the interaction with the plant requires specialization on the part of the microbe. Generalist microbes (squares and circles) tend to have larger genomes, enabling them to occupy different environmental niches and plant hosts, or to exist as facultative endosymbionts or opportunistic pathogens, depending on environmental circumstances. Niche adaptation requires genomic specialization, often via genome reduction (red triangles). The resulting LEANOMEs limit the fitness of a given organism to fulfil multiple roles, or even occupy different hosts, but offer potential tools for synthetic biology approaches to optimize plant–microbe interactions.

Relationships between plants and microbes comprise both fungal and bacterial interactions and can be categorized in various ways, primarily based on location and relationship to the plant, summarized in Figure1. The distinction between free-living soil bacteria, the rhizosphere population and endosymbionts of a plant host may be a true continuum, with microbes able to move between the soil, the root zone and the root, and definition influenced by both theory and methodology. By contrast, the nature of the interaction requires specialization on the part of the microbe, and there is a gradient from obligate pathogen, to opportunistic pathogen, to parasite/commensal, to facultative endosymbiont, to obligate endosymbiont, to plastid, and ultimately to organelle. Some microbes are generalists, for example able to exist as opportunistic pathogen, commensal and facultative endosymbionts, depending on the environmental circumstances; however, niche adaptation requires genomic specialization, limiting the fitness of a given organism to fulfil multiple roles. Adaptation to life within a plant, to the exclusion of the ability to exist in the competitive environment of the soil, is a specialization of great interest and relatively little study. Furthermore, plants have evolved in the presence of these complex microbial communities, yet ‘our knowledge of how this ‘symbiome’ influences host evolution, and development is woefully inadequate’ (Hirsch, 2004). In this context, we are especially interested in those microbes that have specialized as beneficial plant endosymbionts, the key mechanisms by which these interactions are maintained and how we might manipulate these relationships to optimize biomass production.

### Quorum sensing and biofilm formation

Cell to cell communication between bacteria occurs via diffusible chemical signals and is collectively known as quorum sensing (QS, Greenberg, 1997). A number of bacterial genes are regulated by QS, including those involved in swarming, virulence and biofilm formation. Biofilms comprise multicellular assemblies of bacteria embedded in a complex extracellular matrix of exopolysaccharides and proteins. The formation of biofilms enables bacterial populations to adhere to environmental surfaces, including plant tissues, and is an intrinsic component of plant–microbe interactions (Ramey *et al.*, 2004). Transposon mutagenesis of plant-associated *Bacillus amyloliquefaciens* ssp. *plantarum* FZB42 identified genes required for bacterial swarming, biofilm formation, root colonization and plant growth promotion in axenic conditions (Budiharjo *et al.*, 2014). Bacteria within biofilms are phenotypically and physiologically distinct from free-living bacteria and demonstrate increased tolerance to antimicrobial compounds (Ramey *et al.*, 2004). The free-living diazotroph *Azospilrillum brasilense* stimulates root proliferation in wheat after forming dense biofilms on the root (Assmus *et al.*, 1995), and *Bacillus subtilis* mutants deficient in biofilm formation are unable to prevent infection of Arabidopsis by *Pseudomonas syringae* (Bais *et al.*, 2004). Mutagenesis of *B. subtilis* strain 6051 resulted in a mutant strain compromised in production of surfactin, a lipopeptide antimicrobial compound. Whereas *B. subtilis* strain 6051 forms biofilms and secretes surfactin to levels estimated to be lethal to *P. syringae*, the mutant was unable to form robust biofilms and was ineffective as a biocontrol (Bais *et al.*, 2004).

### Plant–microbe signalling

Plant–microbe signalling in the soil occurs by means of chemical interaction, with both partners actively involved. Plants manipulate their interactions with the soil and soil microbes, at significant carbon cost, via rhizodeposition of diverse compounds from the roots. Rhizodeposition comprises root cap and border cells, mucilage, soluble root exudates, volatile organic carbon and the carbon lost to symbionts and through cell and tissue death (Jones *et al.*, 2009). Rhizodeposits, particularly the mucilage and root exudates, modulate the bacterial composition of the rhizosphere around the growing root (Dennis *et al.*, 2010). Different plants attract different populations of prokaryotes and eukaryotes to their rhizosphere (Turner *et al.*, 2013). Amino acids and carbohydrates released by the root may act as chemo-attractants, thereby accounting for the higher numbers of certain bacteria in the rhizosphere as compared to bulk soil (Bacilio-Jiménez *et al*., 2003); however, a range of signalling molecules are required for the more subtle interactions. Differential gene expression in *Pseudomonas aeruginosa* strain PAO1 was observed in response to the root exudates of two different varieties of sugar beet, including previously uncharacterized genes associated with rhizosphere competition and bacterial colonization (Mark *et al.*, 2005). Reciprocal inoculation experiments of *B. subtilis* N11 (isolated from banana rhizosphere) and *B. amyloliquefaciens* SQR9 (isolated from cucumber rhizosphere) on banana and cucumber indicate more efficient colonization of the native host by bacteria from the rhizosphere of the two plants. Analysis of the root exudates of the two plants indicated components that induced both chemotaxis and biofilm formation in the native bacteria, but only one or the other in the non-native strain (Zhang *et al.*, 2014).

Plants inhibit neighbouring plants, herbivorous eukaryotes, and soil bacteria via chemical signalling from root exudates. A range of antimicrobial products are exuded in the root tip mucilage, providing a defensive zone around the meristematic and elongating root cells (Walker *et al.*, 2003). Certain legumes exude the compound canavanine, which is structurally similar to arginine, from their roots. If ingested and incorporated into nascent proteins in place of arginine, canavanine results in structurally aberrant proteins. While canavanine is toxic to many soil bacteria, certain rhizobial strains are able to detoxify canavanine and so are presumably advantaged relative to other bacteria in the rhizosphere of legume roots (Cai *et al.*, 2009). Furthermore, the plant transcriptome and proteome respond to bacterial quorum sensing signals (QSS) by pathogens and symbionts, with protein changes specific to the QSS structure and concentration (reviewed by Mathesius, 2009).

As demonstrated by these examples, QS and biofilm formation represent key targets for bacterial manipulation to optimize plant production.

## Bacterial endophytes

In addition to soil and rhizosphere communities, large and diverse populations of microbes live within plants without causing signs of disease and are broadly termed endophytes. Bacterial endophytes reside within specific plants tissues, either inside the host cells or in the intracellular fluids, and have been isolated from all plant tissues (Rosenblueth and Martínez-Romero, 2006). They can be considered to sit at the benign end of the spectrum between mutualists and pathogens (Hirsch, 2004). These ancient relationships are not only fascinating from an evolutionary perspective, but are potentially of great value for sustainable plant production if these relationships can be understood and exploited.

The majority of endophytes are widely considered to represent a subset of soil bacteria which must colonize the plant without triggering the host defence response. Thus, they must exist in both free-living and endophytic states. In order to transition from the soil to the plant, the bacteria must first demonstrate rhizosphere competence and attachment to the root, followed by establishment in the host plant (Compant *et al.*, 2010). Once inside the plant, endophytes may be either extracellular or intracellular, surrounded by a host membrane. Both motility and secretion of various cellulases and pectinases are necessary attributes of bacteria transitioning from free-living to endophytic lifestyles (Reinhold-Hurek and Hurek, 2011). Endophytic bacteria do not create detrimental effects or cellular damage to the plant. Endophytic bacteria usually have lower population densities in the host plant tissues compared with pathogens, and this may be one method by which they evade the plant defences. There are however, reports of endophytic bacteria colonizing the host tissue internally, sometimes in high numbers, without damaging the host or eliciting symptoms of plant disease (Zinniel, 2002). Far from simply evading the attention of the plant, beneficial endophytes act in part by triggering the plant induced systemic resistance (ISR) towards pathogenic bacteria (reviewed by Kloepper and Ryu, 2006). In order to exploit beneficial bacteria to optimize biomass production, a far deeper understanding of both the individual components and their interactions is required.

Bacterial endophytes have been isolated from virtually all plants studied (Ryan *et al.*, 2008) including a number of potential bioenergy crops (summarized in Table1). This is almost certainly a considerable under-representation of the true diversity of endophytes within these species as many laboratories seek to isolate specific strains of interest rather than the full diversity present. Since the advent of next-generation sequencing, there has been a shift away from targeted isolation of small numbers of microbes towards large-scale projects aimed at sequencing the entire microbial population within an environmental niche. Metagenomic studies on energy crop species have not yet been reported, but in the same way that the human metagenome revealed the full extent of microbial associations with mammals (Zhao, 2010), diverse communities of endophytic bacteria have been identified in plant microbiome projects (Lundberg *et al.*, 2012; Sessitsch *et al.*, 2012). In terms of understanding biological function, a range of molecular tools are of use, including complete genome sequences (Table1), transcriptomics (Mark *et al.*, 2005; Shidore *et al.*, 2012; Straub *et al.*, 2013b; Zuccaro *et al.*, 2011), proteomics (Lery *et al.*, 2011; Mathesius, 2009), and fluorescent tagging and localization studies (Compant *et al.*, 2010; Reinhold-Hurek and Hurek, 2011; Ryan *et al.*, 2008).

**Table 1:** Reported bacterial endophytes isolated from bioenergy crops

Bioenergy crop	Endophytic bacteria	References
Sorghum	Herbaspirillum seropedicae*	Baldani *et al*. (1986) *Pedrosa et al. (2011)
Pennisetum	*Azospirillum brasilense*, *Gluconacetobacter diazotrophicus*, *Gluconacetobacter liquefaciens*, *Gluconacetobacter sacchari*, *Burkholderia silvatlantica*, *Klebsiella* sp., *Enterobacter cloacae and Enterobacter oryzae*	Videira *et al*. (2012)
	*Herbaspirillum*-like *Herbaspirillum frisingense sp., Herbaspirillum seropedicae*	Kirchhof *et al*. (1997) Kirchhof *et al*. (2001) Olivares *et al*. (1996)
Sugarcane	*Gluconacetobacter diazotrophicus* (*syn. Acetobacter diazotrophicus*)*	Gillis (1989), Dong *et al*. (1994) *Berlatan et al. (2009)
	*Burkholdeira*, *Pantoea*, *Pseudomonas*, *Microbacterium*	Mendes *et al*. (2007)
	*Citrobacter*, *Enterobacter*, *Pantoea*, *Klebsiella*, *Erwinia*, *Brevibacillus*, *Staphylococcus*, *Curtobacterium*, *Pseudomonas* sp.	Magnani *et al*. (2010)
	*Bacillus*, *Paenibacillus*, *Brevibacillus*, *Cohnella*	Raton *et al*. (2012)
	*Burkholdeira australis*	Paungfoo-Lonhienne *et al*. (2014)
	Herbaspirillum seropedicae*, *Herbaspirillum rubrisubalbicans*	Olivares *et al*. (1996) *Pedrosa et al. (2011)
Miscanthus	*Azospirillum*-like, *Azospirillum lipoferum*-like, *Herbaspirillum*-like	Kirchhof *et al*. (1997)
	*Azospirillum doereinerae* sp. nov. GSF71	Eckert et al. (2001)
	*Herbaspirillum frisingense* sp.	Kirchhof *et al*. (2001) and Straub et al. (2013a)
	*Clostridium* spp.	Miyamoto *et al*. (2004)
Poplar	*Methylobacterium populi*, *Pseudomonas* sp.	Van Aken (2004), (summarised in van der Lelie, 2009)
	*Enterobacter* sp 638*	Taghavi et al. (2009)
	*P. putida* W619	*Taghavi et al. (2010)
	*Serratia proteamaculans* 568	
	*Stenotrophomonas maltophilia* R551-3	
	*Burkholderia vietnamiensis*	Doty *et al*. (2009)
	*Pantoea* sp.	
	*Pseudomonas graminis*	
	*Rahnella* sp. CDC 2987-79	
	*Enterobacter* sp. YRL01	
	*Burkholderia* sp. H801	
	*Acinetobacter calcoaceticus*	
Willow	*Acinetobacter* sp. *PHD-4*	Doty *et al*. (2009)
	*Herbaspirillum*	
	*Stenotrophomonas* sp. *LQX-11*	
	*Sphingomonas yanoikuyae*	
	*Pseudomonas* sp. *H9zhy*	
	*Sphingomonas* sp. *ZnH-1*	
	*Pseudomonas* sp. *H9zhy*	
	*Sphingomonas yanoikuyae*	
	*Sphingomonas* sp. *ZnH-1*	
	*Pseudomonas* sp. *WAI-21*	
	Pantoea agglomerans*	*Gan et al. (2014)
	Staphylococcus haemolyticus*	
	*Pseudomonas* sp.*	
	Microbacterium oleivorans*	
	Micrococcus luteus*	
	Micrococcus luteus*	
	Janthinobacterium lividum*	
	*Stenotrophomonas* sp.*	
	*Delftia* sp.*	
	Micrococcus luteus*	
	*Sphingomonas* sp.*	
	*Exiguobacterium* sp.*	
	*Pseudomonas* sp.*	

* Indicates a published genome sequence is available, and the corresponding reference.

## Plant benefits and near-term exploitation

Bacteria that convey benefits to the plant are collectively termed plant growth promoting bacteria (PGPBs). PGPBs may either be rhizobacteria (PGPRs) or colonize plant roots to become endophytes, with a number of species moving between the two states (Compant *et al.*, 2010). There is a great potential for optimizing biomass production through the application of plant-associated bacteria, as evidenced by a 55% biomass increase in poplar cuttings 17 weeks after inoculation with *Enterobacter* sp. Strain 638 (Rogers *et al.*, 2012). PGPBs are diverse in their modes of action, including production of phytohormones, nitrogen acquisition, mobilization or enhanced uptake of soil minerals such as phosphorus, plant protection and control of pathogens. These benefits have not always been realized when applied to field situations, potentially due to insufficient rhizosphere or plant colonization (Compant *et al.*, 2010). *Azospirillum,* in particular, has been studied extensively both as a PGPR and as an endophyte and is used as a commercial inoculant to improve yields and/or reduce expensive fertilizer use (Baldani *et al.*, 1987; Bashan, 1998; Hungria *et al.*, 2010; Okon and Itzigsohn, 1995). However, the ability of endophytes to live within plant tissues represents a unique niche, increasing the potential for successful application to boost crop production, and presumably requiring genomic specialization (Hardoim *et al.*, 2008).

### Phytohormone signalling

The roles of phytohormones in plant growth and development are fundamental, diverse and complex, combining both default developmental pathways and dynamic responses to the environment (reviewed recently by Durbak *et al.*, 2012). It is maybe unsurprising then that phytohormones are key components of plant–microbe interactions. Certain bacteria have the ability to produce phytohormones including indole-3-acetic acid (IAA, an auxin), gibberellin (GA) and cytokinin (CK) (Bottini *et al.* 2004; Tsavkelova *et al.* 2006). It has been theorized that phytohormones could be used as signalling molecules between bacteria and host, and the existing crosstalk between IAA and ethylene biosynthesis exploited as a means of communication (Spaepen *et al.* 2007; Yuan *et al.* 2008). Furthermore, bacteria can also influence and regulate phytohormone production by the plant.

Inoculation of Miscanthus seedlings with *Herbaspirillum frisingense* GSF30^T^, a temperate grass endophyte, promoted root and shoot growth; transcriptome analyses revealed regulation of jasmonate and ethylene signalling, indicating that the promotion of plant growth is modulated by phytohormone activity (Straub *et al.*, 2013b). Of eleven different endophytic bacterial strains isolated from sweet potato, the cuttings inoculated with bacterial strains that produced indole acetic acid (IAA) and auxin produced roots first and grew more rapidly than uninoculated cuttings (Khan and Doty, 2009). *Herbaspirillum frisingense* GSF30^T^ was demonstrated to produce IAA in culture (Rothballer *et al.*, 2008), and auxin was concluded to be the likely mechanism behind the increase in seedling growth of wheat plants inoculated with *B. subtilis* (Egorshina *et al.*, 2011). *Azospirillum* spp. are considered to increase plant growth primarily via root stimulation by auxin, with nitrogen fixation and other production of phytohormones playing lesser roles (Steenhoudt and Vanderleyden, 2000). These effects may well be applicable in field situations, for example *Azopspirillum* sp. strain B510, isolated from surface-sterilized stems of rice, significantly increased tiller number and yield of paddy field-grown rice plants following re-inoculation of seedlings (Isawa *et al.*, 2010) while three *Pseudomonas* strains enhanced growth and spike length in wheat in both laboratory and field conditions (Iqbal and Hasnain, 2013). These effects were attributed to phytohormone production rather than nitrogen fixation in both cases.

Ethylene plays an important role in both normal plant development and plant stress response. Ethylene synthesis is highly sensitive to environmental stimuli including light, temperature and other phytohormones, with production increased in response to a variety of biotic and abiotic stresses (Abeles *et al.*, 1992). Bacterial species with the ability to produce 1-aminocyclopropane-1-carboxylate (ACC) deaminase, for example *Burkholderia* spp., can degrade excess amounts of ACC (the direct precursor to ethylene) producing nitrogen and energy as a by-product, reducing the stress response and promoting growth (Onofre-Lemus *et al.*, 2009). Ethylene levels in the plant may be regulated by cleaving ACC or inhibiting its production; in either case, bacterial efficiency increases in close proximity to the plant cells in which ethylene biosynthesis occurs (Hardoim *et al.*, 2008). Bacteria with ACC deaminase activity frequently provide a range of other benefits and have been postulated to be major forerunners in the transition from chemicals to bacterial plant growth promotion in agricultural systems (Glick, 2014).

### Nutrient acquisition

A number of bacterial endophytes have the ability to form symbioses with plants and to fix bio-available nitrogen within unspecialized tissues of the host plant, that is in the absence of nodulation as seen in the legume–rhizobia interaction. For example, Cyanobacteria can form associations with a range of plants from different clades including Gunnera, cycads, lichens and Azolla (Santi *et al.*, 2013) and form heterocysts; specialized structures creating a microaerophilic environment suitable for nitrogen fixation with the nitrogenase enzyme (Berman-Frank *et al.*, 2003).

Several diazotrophic bacterial species have been repeatedly identified as being associated with, or as bacterial endophytes of, *Saccharum* (sugar cane) in Brazil. These species include *Gluconacetobacter diazotrophicus* (formerly *Acetobacter diazotrophicus*), *Azospirillum amazonense*, *A. brasilense*, *Herbaspirillum seropedicae* and *Herbaspirillum rubrisubalbicans* (formerly *Pseudomonas rubrisubalbicans*, Kirchhof *et al.*, 1998; Monteiro *et al.*, 2012a). *Gluconacetobacter diazotrophicus* can be endophytic in *Saccharum* and has been identified in electron microscopic studies using immunogold labelling techniques (James *et al.*, 1994). Both *G*. *diazotrophicus* and the mild plant pathogen *Herbaspirillum* spp. have been recorded in high numbers in sugar cane roots, stems and leaves (James and Olivares, 1998; Olivares *et al.*, 1996). *Herbaspirillum seropedicae* populations are reduced in bulk soil compared with plant-associated populations (Baldani *et al.*, 1992) suggesting the species is particularly suited to an endophytic life. *Herbaspirillum rubrisubalbicans* also has the ability to colonize sugar cane endophytically (James *et al.*, 1997). These species of diazotrophic bacteria are likely to be key contributors to the significant biological nitrogen fixation (BNF) that has been observed in field experiments using nitrogen balance and nitrogen isotope dilution techniques in Brazilian sugar cane (Baldani and Baldani, 2005; Boddey *et al.*, 1991; Döbereiner *et al*., 1993; James, 2000).

Videira *et al*., (2012), used semisolid media to culture bacteria from fresh tissue of two genotypes of *Pennisetum purpureum* to investigate possible nitrogen-fixing bacterial populations. The culturable diazotrophic bacterial population colonizing these plants varied from 102 to 106 bacteria/g fresh tissues. Diazotrophs identified belonging to the genera *Gluconacetobacter*, *Azospirillum* and *Enterobacter* colonized the plant tissues of both genotypes, similar to those found in Brazilian sugar cane and Miscanthus grown in Illinois (Davis *et al.*, 2010), indicating that these relationships are common to temperate and tropical systems.

In rice and maize, BNF contribution is similarly derived from a number of different species including members of *Azospirillum*, *Azoarcus*, *Herbaspirillum*, *Bacillus*, *Enterobacter*, *Klebsiella* and *Pseudomonas* (Boddey *et al.*, 1995; Hurek *et al.*, 1994; Kirchhof *et al.*, 1998; Monteiro *et al.*, 2012a). In field experiments using wild rice, grain yields increased to the equivalent of using an additional nitrogen fertilizer application of 40 kg N/ha following inoculation with *Herbaspirillum seropedicea* (Baldani *et al.*, 2000; Pereira and Baldani, 1995). In another study, up to 30% of the total nitrogen accumulated in rice plants was derived from BNF, again demonstrating the potential gains to be made from bacterial associations (Malik *et al.*, 1997). Sixteen percent of plant nitrogen in field-grown Miscanthus plants was estimated to be derived from BNF, despite nonlimiting soil nitrogen (Keymer and Kent, 2013). However, *A. diazotrophicus* colonization of sugar cane is inhibited by high N-fertilization (Fuentes-Ramirez *et al*., 1999), and exogenous nitrogen fertilizer has been demonstrated to reduce the number of diazotrophic endophytes cultured from sugar cane (Pariona-Llanos *et al.*, 2010). In Brazil, both rhizosphere and endophyte populations were demonstrated to be altered following the practice of adding vinasse, a concentrated by-product of the sugar extraction process rich in nutrients, back to soils to fertilize sugar cane (Leite *et al.*, 2014). These data indicate that diazotrophic relationships may not be retained by the plant in conditions where they are not conveying a benefit in terms of nitrogen availability. Interestingly, the ability to fix nitrogen of some diazotrophic *Herbaspirillum* strains has been documented in wild rice, but the results were not replicated in the same experiment with cultivated rice (Elbeltagy *et al.*, 2001; Kirchhof *et al.*, 2001). Genomic comparison of wild and cultivated rice should yield insights into the plant components required for successful plant–diazotroph interactions.

Finally, plants may obtain nitrogen from associated bacteria via active release of amino acids by diazotrophs. At least one, and up to four, amino acids were released from each of 22 strains of diazotrophic rhizobacteria isolated from sugar cane and grown on media free of combined-N. The excretion of amino acids was correlated with nitrogenase activity and included methionine and ornithine, both precursors of ethylene (de Oliveira *et al.*, 2011).

### Plant protection and biocontrol

A range of essential microbial components, collectively termed microbe-associated molecular patterns (MAMPs) are recognized by plants and act as elicitors, triggering a generalized MAMP-triggered immunity (MTI). Although commonly described in the context of pathogenicity, MAMPs are conserved among nonpathogens including endophytes. MTI responses include the production of molecules such as reactive oxygen and nitrogen species, which act in signalling and as antimicrobial compounds (reviewed by Newman *et al.*, 2013). Induction of systemic plant resistance by either rhizosphere or endophytic bacteria is independent of salicylic acid accumulation and pathogen-related protein induction and is termed induced systematic resistance (ISR) to distinguish the response from systemic acquired resistance (SAR), which is triggered by pathogens (van Loon *et al.*, 1998; Pieterse, 1998). Pre-inoculation of Arabidopsis seedlings with two closely related strains of *Streptomyces* sp. protected the plants from disease symptoms following subsequent challenge by *Erwinia caratovora* while endophyte-free plants succumbed to rot within 5 days. Despite morphological and taxonomic similarity of the two strains, gene induction in Arabidopsis was specific to each of the two strains following inoculation, indicating ISR induction by one and SAR induction by the other. The host response is therefore fine-tuned to respond to different bacterial signals, further indicated through induction of ISR by bacterial exudate grown on a complex medium, and SAR induction by exudate of the same strain grown on minimal medium (Conn *et al.*, 2008). Furthermore, there is an evidence that pathogen infection itself triggers plant recruitment of beneficial rhizosphere bacteria. Infection of Arabidopsis by *P. syringae* induced a malic acid (MA) transporter, in turn led to an increase of MA in the rhizosphere. *Bacillus subtilis*, a beneficial rhizobacteria, numbers increased in response to MA, and stimulated ISR in the plant, thereby restricting the effect of the pathogen (Lakshmanan *et al.*, 2012).

The role of endophytes in eliciting plant defence in energy crops is not yet well studied, although *G. diazotrophicus* has been demonstrated to elicit a defence response against a plant pathogen in sugar cane (Arencibia *et al.*, 2006). Further analysis of the signalling that occurs following endophytic and pathogenic inoculation, such as the proteomic analysis conducted in sugar cane by Lery *et al*. (2011), will indicate whether similar or divergent mechanisms are involved in these crops in comparison to Arabidopsis. In this study, host genotype-specific responses were observed in the proteome of *A. diazotrophicus*, with one strain of sugar cane expressing proteins involved in root colonization, while the other elicited a strong defence, preventing a successful interaction. It is highly likely that the rhizosphere and endophytic populations will vary between crops grown at different geographies and that the resulting interactions will be largely specific to the plant and microbial strains as well as the environmental conditions. For energy crops, in particular, it will be of importance to design experiments to understand these interactions in controlled environments approximating those in the field.

An alternative mechanism of plant protection by rhizosphere and endophytic bacteria is the production of antimicrobial compounds. Rosmarinic acid, which demonstrated potent antimicrobial activity against a range of soil borne microorganisms, was induced in the exudates of sweet basil hairy root cultures following challenge by *Pythium ultimum* (Bais *et al.*, 2002). Endophytic actinobacteria, in particular, have been a rich source of novel bioactive compounds, including antibiotics, antifungals and antitumour compounds with great potential for exploitation (summarized in Qin *et al.*, 2011). In addition to the production of specific antimicrobial products, endophytic bacteria inhibit pathogenic QS, thereby inhibiting communication and biofilm formation, and hence virulence, without suppressing bacterial growth. Cell-free lysates from endophytic bacteria were demonstrated to degrade QS molecules and suppressed biofilm formation in *P. aeruginosa* PAO1 (Rajesh and Ravishankar, 2013). Thus, endophytic bacteria can protect the host against pathogens which have evolved resistance to the plant defences. This ‘quorum quenching’ is of great interest as an alternative antivirulence approach to tackling drug-resistant bacteria as it does not induce selective pressure for developing antibiotic resistance (Kusari *et al.*, 2014).

### Abiotic stress tolerance

In an era of changing climates, there are obvious advantages to developing crops with tolerance to abiotic stresses such as drought and salinity. In the case of perennial energy crops, which are to be grown on marginal land, resilience to a wider range of stresses is essential. Such crops must overwinter annually and tolerate the climatic conditions over multiple seasons, perhaps for a decade or more. They must generate high-biomass yields on land unfit for food production; for example due to low or erratic rainfall, salinity or heavy metal pollution. While abiotic stress tolerance may be conferred by the plant genome, relationships with microbes can also provide improved tolerance to, or protection from, numerous abiotic stresses.

*Burkholderia phytofirmans* strain PsJN has a wide host spectrum, including wheat, maize and grapevine, and has been implicated in a range of beneficial abiotic stress tolerance. Photosynthetic rate, water-use efficiency and chlorophyll content of wheat inoculated with *B. phytofirmans* PsJN were improved with respect to controls under field conditions, ultimately resulting in increased grain yield (Naveed *et al*.,2014a). In maize shoot and root biomass, leaf area and photosynthetic efficiency was higher in droughted plants inoculated with both *B. phytofirmans* and *Enterobacter* sp. FD17 with respect to controls. *Burkholderia phytofirmans* offered more efficient protection against drought, indicating that physiological responses to endophyte inoculation are specific to the plant and microbial genotypes (Naveed *et al.*, 2014b). *Burkholderia phytofirmans* PsJN induces resistance to grey mould and increases tolerance to low nonfreezing temperatures in grapevines. Following growth at 4 °C, more rapid and greater up-regulation of the plant stress-related gene transcripts and metabolites was observed in the plant in the presence of the bacteria, indicating a priming effect of the endophyte (Theocharis *et al.*, 2012). *Burkholderia phytofirmans* PsJN has been demonstrated to colonize and promote the growth of switchgrass under glasshouse conditions (Kim *et al.*, 2012), suggesting it may be an excellent candidate for bioenergy production enhancement.

A number of other endophytes have also been shown to confer tolerance against abiotic stresses to plants. Miscanthus was demonstrated to be more tolerant to salinity following inoculation with an anaerobic diazotroph *Clostridium* and a nondiazotrophic *Enterobacter* sp. Despite an initial slight retardation in growth with respect to uninoculated plants, inoculated plants were larger than the controls following continuous growth on 100 mM NaCl (Ye *et al.*, 2005). *Gluconacetobacter diazotrophicus* has a wide host range and is a common endophyte of sugar cane, where it tolerates high sucrose concentrations. Expression of levansucrase is required by the bacteria to hydrolyse sucrose to glucose and fructose for transport into the cell and subsequent metabolism. Disruption of the gene encoding levansucrase results in decreased levansucrase production, decreased tolerance to desiccation and decreased tolerance to NaCl, indicating that levansucrase may act as an osmoprotectant (Velázquez-Hernández *et al*., 2011). Collectively, the physical and chemical bacteria-induced changes resulting in plant abiotic stress tolerance have been termed ‘induced systemic tolerance’ (IST) (Yang *et al.*, 2009).

Screening plants growing in extreme environments is a promising approach to isolating novel endophytes for application in energy crops to be grown under marginal conditions. Seventeen of 20 bacteria, predominantly *Bacillus* sp., isolated from halophyte and salt-tolerant plant species showed growth in culture on 7.5% NaCl, with all but two tolerating up to 10% NaCl (Arora *et al.*, 2014). The high frequency of halotolerance among the endophytes of plants growing in saline environments suggests that the plant may more readily recruit stress tolerance from a diverse bacterial population than develop innate tolerance via adaptation of the plant genome. If this is the case, this has broad implications for energy crop breeding and agronomy.

### Phytoremediation

A potential dual benefit of an energy crop plantation is the possibility to use a biomass crop for phytoremediation of a contaminated site. In addition to harvesting a biomass crop for use as an industrial feedstock for fuel or renewable product production, an energy crop can be used to decontaminate land unsuitable for food production in order to bring it back into use. In pot experiments, endophytic *Bacillus* sp. SLS18 increased biomass of Sorghum grown in either manganese or cadmium. Similar effects were also observed in two dicotyledonous species, again indicating broad applicability in terms of host range (Luo *et al.*, 2012). Remediation of both organic compounds and toxic metals is possible, each dependent on effective plant–microbe interactions. Phytoremediation uses plants to clean up toxic soils, whereas the process phytoextraction uses species which uptake and accumulate trace element concentrating the pollutants in their tissues and out of the soil. Fast-growing high-biomass plants including *Populus trichocarpa* and *Salix* spp. are often used for phytoextraction, and the process is enhanced by inoculating the plants with bacterial endophytes. The plant provides a biological niche to support higher microbial densities of microbial populations or consortia able to successively transform contaminants. Contaminants may either be neutralized or stored in the plant and harvested, thereby remediating the soil. However, specialist applications or residual metal recovery may be required to prevent future recontamination from the biomass. Endophytes including *Burkholderia cepacia* have been demonstrated to both increase the efficiency of the remediation and also boost biomass production in the host (Weyens *et al.*, 2009a,b).

Bioprospecting for endophytes in a range of hostile environments may be a route to developing energy crops tolerant to growth on contaminated soils. A high rate of cadmium tolerance was observed in endophytes isolated from the seed of tobacco plants grown with exposure to cadmium. When inoculated with these endophytes, tobacco plants accumulated increased biomass in both the presence and the absence of cadmium. Moreover, cadmium was accumulated to a greater concentration in endophyte inoculated plants (Mastretta *et al.*, 2009). Targeted screening of plants from extreme environments (saline, droughted, contaminated etc.) may yield a wealth of novel microbes with adaptive traits of interest for application in energy crops for growth on marginal land.

A summary of beneficial plant–microbe interactions and near-term applications is shown in Table2. The increasing number of patents in this area is indicative of the opportunities that these beneficial organisms present (Mei and Flinn, 2010). In practice, countless beneficial bacteria have yet to be isolated and identified, and of those that have, many confer multiple benefits to plants. Wide-spectrum benefits may be conferred to plants via associations with microbes, including both rhizosphere bacteria and endophytes. In addition to promoting plant growth via phytohormone production, PGPRs may further augment plant immunity and elicit both IST and nutrient uptake (Yang *et al.*, 2009). In a study of 102 bacteria associated with sugar cane roots, 74 were able to fix nitrogen and 77 were able to solubilize phosphate, all 102 produced IAA to at least some degree, 50 were positive for the production of the QS molecule *N*-acyl homoserine lactone (AHL), and 33 isolates were positive for all four tests. Twenty-seven isolates were further tested for salinity tolerance (Leite *et al.*, 2014). Harnessing these benefits to promote biomass crop growth will require a combination of detailed understanding of the component microbes and their interactions with plants, for example by mutagenesis (Rouws *et al.*, 2008), and also long-term field studies to determine the factors regulating microbial populations in the rhizosphere and soil. A near-term application is the development of strains to be used as seed coatings, biofertilizers and biopesticides.

**Table 2:** Summary of beneficial plant–microbe interactions and near-term applications

Activity	Application	Priority
Phytohormone production	Plant growth promotion	Develop synthetic consortia for use as yield boosting agents
Biological nitrogen fixation/phosphate soulbilization	Biofertilizer	Identify novel strains and elucidate host–microbe specificity mechanisms
Plant protection	Biocontrol	Screening of endophyte collections for antimicrobial properties and plant defence induction
Abiotic stress tolerance	Boost plant biomass on marginal land	Bioprospecting for endophytes of plants growing under extreme conditions, for example drought, cold and salinity
Phytoremediation	Remediation of contaminated land	Bioprospecting for endophytes of plants growing on a range of contaminated sites
Endophytic specialization	Novel pathways and reduced genomes for synthetic applications	Genome analysis of endophytic and closely related species and development of molecular parts and devices libraries

## Synthetic bacterial populations

**Figure 2:**
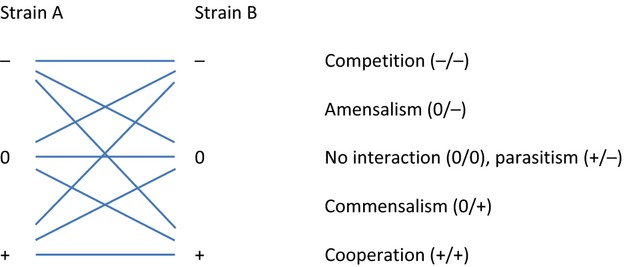
The possible six motifs of microbial interactions between two bacterial strains (developed from Großkopf and Soyer, 2014).

An alternative to identifying single strain isolates with a range of plant benefits is the potential for developing synthetic bacterial communities for application to crops. There is currently an increasing interest in generating synthetic consortia of two or more bacteria to address questions of community-level functions and properties. Previous reports, that competition between bacteria is common, were based on pairwise experiments of isolated strains (Foster and Bell, 2012); however, results of studies to date indicate that even paired interactions are complex in terms of function and stability. One strain may provide a metabolic effect with a negative, neutral or positive effect on the other; six motifs of microbial interactions are possible from a combination of two bacteria (Figure2), with this complexity rapidly increasing to 729 interaction states for a community of three strains, and 531 441 interaction states for a community of four strains (Großkopf and Soyer, 2014). In reality, even simple microbial communities sampled from the natural environment contain far higher numbers of individuals, with seeding of new strains possible at all times. Environmental conditions such as pH, temperature and nutrient availability will affect growth rates of the individuals (Goldfarb *et al.*, 2011), all of which change over time with bacterial growth. There may be a high or a low rate of seeding in different populations, with certain systems, such as anaerobic digesters, providing a relatively consistent environment in which a bacterial community may stabilize (Werner *et al.*, 2011). In comparison to the dynamic environment of the soil, the internal tissues of a plant are likely to provide a relative stable environment for a population of bacteria adapted to endophytic life. The challenge then is not to attempt to control this diversity at a species level, but to develop consortia with resilient functionality in terms of plant growth promotion.

## Reduced/specialized microbial genomes

**Figure 3:**
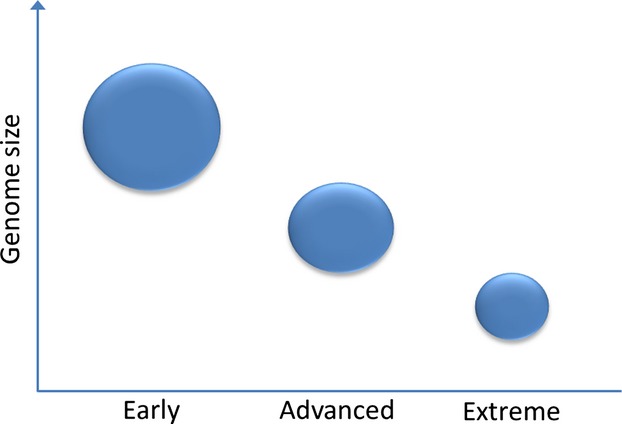
Bacterial genome size associated with the stage of intracellular host adaptation. Early = facultative intracellular; advanced = obligate intracellular; extreme = obligate intracellular mutualistic (cooperative). (adapted from Toft and Andersson, 2010).

Although much attention has been paid to gain of function mutations, loss of function mutation can occur at high frequency and plays an important role in adaptation. Under selection, substantial adaptation to new environments, via altered metabolism, can be achieved through loss of function mutations (Hottes *et al.*, 2013). Bacteria that have evolved an obligate endosymbiotic relationship with their host are known to have undergone genome reduction during host adaptation stages compared with free living and often pathogenic-related species (Figure3). Statistical analyses confirm that among the γ-proteobacteria genome size is inversely correlated to the intracellular stage of host adaptation (Toft and Andersson, 2010). The genome of *H. seropedicae* SmR1, a specialized endophyte of tropical grasses, is composed of a circular chromosome of just over 5.5 Mbp, whereas the genome of the pathogen *H. rubrisubalbicans* M1, causal agent of mottled stripe disease and red stripe disease, was estimated to be over 50 Mbp. (Monteiro *et al.*, 2012b; Pedrosa, 2011). In addition to differences between these species in a range of molecular factors potentially involved in colonization, almost 40% of the suppressive subtractive hybridization library of *H. rubrisubalbicans M1 contained* mobile elements [insertional sequences (IS)] compared with zero IS being identified in *H. seropedicae* (Monteiro *et al.*, 2012b). These mobile elements are known to exert plasticity on the bacterial genome and facilitate activation or inactivation of genes resulting in altering the metabolic network and conferring an evolutionary selective advantage in highly variable environments. The absence of mobile elements and the small genome size of *H. seropedicae* SmR1, seemingly reflect its specialized endophytic lifestyle with tropical grasses. Interestingly, *B. phytofirmans* PsJN is an endophyte that successfully colonizes potato, tomato, onion roots, maize, barley and agricultural soil and has a genome of 8.2 Mbp. In this case, the large genome is not associated with pathogenicity, but harbours a broad range of physiological functions that facilitate *B. phytofermans* PsJN ability to colonize such a wide variety of plant species (Mitter *et al.*, 2013). Again, this demonstrates the relationship between genome size and host specialization. Over evolutionary time, as the bacteria–host plant relationship moves towards obligate mutualism, the symbiont moves towards a low-evolutionary adaptive genome (LEANOME) that is both small and devoid of mobile elements (Figure1). The minimization of genetic and metabolic redundancy is influenced by the metabolite-rich cellular environment, which lowers the selective pressure to maintain metabolic networks leading towards eventual gene loss (Moran *et al.*, 2009; Toft and Andersson, 2010).

The generation of, IS-free *E. coli* MDS42 has been shown to generate microbial chassis with reduced ability to evolve and improved maintenance of unstable genetic constructs (Umenhoffer *et al.*, 2010). On this basis, endophytes that are at an advanced stage of mutualism with host plants may provide stable chassis for the introduction of new genetic cargo and dynamic circuits and signal transduction pathways as a result of their naturally evolved LEANOMEs; reduced genome size and low-evolutionary adaptability. Bacterial endophytes, in particular those with reduced genomes adapted to living their entire life cycles in planta, offer a wealth of role diversity and potential to understand and manipulate these mechanisms.

## Synthetic approaches

In the longer term genetic and genomic analysis of selected strains will enable targeted modification of bacterial genomes in order to confer improved benefits to the crop (Straub *et al.*, 2013a). Major advances are being made in synthetic biology, with whole pathways engineered into or out of bacterial genomes to modify metabolic function (Reviewed in Jarboe *et al.*, 2010). Bacterial endophytes are of particular interest as sources of traits, genes and pathways which confer benefit to the plant host and also to understand and exploit the genomic adaptations required to live within a plant without causing disease or succumbing to the plant defence systems.

Synthetic biology has been defined as ‘the engineering of biology: the synthesis of complex, biologically based (or inspired) systems, which display functions that do not exist in nature’ (EUR 21796). This engineering perspective may be applied at all levels of the hierarchy of biological structures—from individual molecules to whole cells, tissues and organisms. In essence, synthetic biology will enable the design of ‘biological systems’ in a rational and systematic way’ (Serrano, 2007). The advent of synthetic biology enables manipulation of genomes beyond the random insertion or reduced transcription of one or two genes. Not only can artificial chromosomes, multiple genes, whole pathways be introduced into new organisms at specific genomic locations, but these genes may be artificially synthesized to incorporate alternative functions or regulation, with genes also being excised completely from a genome, leaving no molecular footprint. Such approaches are accelerating rapidly in a small number of bacterial and yeast species, for example metabolic engineering of fermentation biocatalysts to produce cost effective biofuels and products (Reviewed in Jarboe *et al.*, 2010); however, their application to plants is not straightforward. Plant–microbe interactions offer a more tractable option, as the microbial partner can be targeted to produce benefits to the plant.

A major target for synthetic biology currently is to engineer BNF in nonlegumes. The identification of a host–endophyte system for crops that is capable of nitrogen fixation offers the potential of both improving crop productivity, while reducing fertilizer inputs that would result in a concomitant lowering of greenhouse gas emissions. However, despite exhaustive efforts, this mutualistic relationship, common to legumes, has not been identified in any agronomically important members of the gramineae, such as rice, wheat or maize. Recent advances in synthetic biology offer the potential to redesign and engineer the nitrogen fixation pathway into non-nitrogen-fixing bacterial hosts that live in close association with one or more of these nutritionally important food crops. Although the majority of known BNF occurs within the root nodules of legumes, this is not unique, for example *Rhizobia* is also capable of inducing formation of nodules with BNF capacity on the roots of *Parasponia*, a nonlegume in the *Cannabaceae* family (Cao *et al.*, 2012). Recently, Temme *et al*. (2012) have targeted the DNA sequence of the nitrogen fixation (*nif*) gene cluster of *Klebsiella oxytoca*. In this landmark study, the entire *nif* gene cluster DNA sequence was systematically ‘refactored’ *in silico* (a software development term meaning that the program code has been rewritten to achieve stability while not compromising functionality, Fowler and Beck, 1999). All native regulation was removed, and the cluster placed under the control of synthetic molecular components, a toolbox of standardized parts and devices with known transcript/lational strength for each gene of the original cluster. All known and unknown regulatory sequences such as ribosomal binding sites, operators, promoters, secondary mRNA structure, and methylation pattern and pause sites in essential genes were removed by changing the codon usage (Temme *et al.*, 2012). The result was the production of a synthetic functional *nif* gene cluster that bore little genetic similarity to the wild-type cluster, thereby enabling orthogonal control through regulatory sensors designed on a separate plasmid. While nitrogen fixation was modest, interestingly, the synthetic BNF pathway also demonstrated nitrogen fixation in the presence of ammonia that would normally inhibit the wild-type activity. The authors have demonstrated the possibility of redesigning complex biosynthetic pathways and placing them under the regulatory control of synthetic sensors and circuits, thereby offering the potential of horizontal transfer to non-N-fixing bacterial species that live in close association with crops. Collaborative research in this area is currently being funded by both the Bill and Melinda Gates Foundation (https://www.ensa.ac.uk/home/) and the National Science Foundation (NSF) of the United States and Biotechnology and Biological Sciences Research Council (BBSRC) of the United Kingdom (http://www.bbsrc.ac.uk/funding/opportunities/2012/ideaslab-nitrogen-improving-on-nature.aspx, http://synbiology.co.uk/designing-crops-of-the-future/). The aim of these projects, respectively, is to engineer new cereal cultivars harbouring the requisite genetic factors for association with nitrogen-fixing bacteria and to engineer both an N-fixing microbe and a model grass to optimize the interaction and deliver maximum nitrogen to the plant. These combined efforts offer the potential of increasing yields of both food and lignocellulosic feedstock in sustainable agricultural systems.

While microbial synthetic biology has been undergoing rapid advancement, efforts in plant synthetic biology and the development of standardized parts and devices for uptake by plant biologists is in its infancy. In part, this may be due to the relatively lower amenability of plant systems towards targeted genome editing using tools such as transcriptional activator-like effector nucleases (TALENs) or clustered regularly interspaced short palindromic repeats (CRISPRs) and their associated proteins. Although our understanding of the double-strand break-repair mechanisms in plants is improving, the rapid advances seen in microbial systems have been confounded by the complexity of gene expression during plant development coupled with the innate recalcitrance of plant genomes to accommodate homologous recombination that would otherwise facilitate routine targeted alleic replacement (reviewed in Sun and Zhao, 2013; Puchta and Fauser, 2014). There have, however, been impressive advancements in this field (Feng *et al.*, 2013) along with the development of a genetic circuit capable of detecting the explosive trinitrotoluene (TNT) that visually reports back a decrease in the production of chlorophyll (Antunes *et al.*, 2011). The synthetic circuit was based on the work by Looger *et al*. (2003) where periplasmic binding proteins (PBP), bacterial chemotactic proteins, where computationally redesigned to accept TNT as a ligand with the complex then binding to a transmembrane histidine kinase resulting in signal transduction to induce expression of β-galactosidase. In the tobacco system, this synthetic signal transduction pathway was adapted to drive expression of GUS or red chlorophyll reductase (*AtRCCR*), with the latter demonstrating de-greening of the plant following exposure to TNT (Antunes *et al.*, 2011).

These advances indicate the possibility of designing synthetic sensors and regulatory circuits which, when coupled together, would enable the controlled induction of bioprocessing enzymes from the endophyte upon stimulation of host signals derived during senescence. From 152 endophytic fungi and 52 endophytic bacteria, 91.7% and 64%, respectively, were found to produce xylanases (Suto *et al.*, 2002). These glycosylhydrolases are required for the deconstruction of plant biomass during biorefining processes and the production of sugar rich syrups for bioconversion into liquid fuels. It is conceivable to computationally design the PBP of bacterial endophytes to accept a specific metabolite(s) that accrue *in planta* during senescence to initiate transcriptional activation of endogenous or recombinant xylanases and/or other biomass processing enzymes such as ferulic acid esterases. Such synthetic plant–microbial systems may be advantageous over senescence promoter driven heterologous expression *in planta* (Buanafina *et al.*, 2008, 2010), in terms of editing a specific location in the microbial genome, providing tightly controlled synthetic signal transduction *in symbiota* by a specific plant-derived metabolite, produced at a specific time, following an environmental cue. Traditional transgenic approaches can often result in obtaining only a few, or aberrant, plant phenotypes possibly arising from ‘leaky’ expression during development, nonspecific genome targeting or subcellular localization. These microbial ‘Trojan horse’ systems are suited to the sustainable agritech production of energy crops. Plant beneficial circuit characteristics (drought/salinity tolerance) may be designed and introduced into endophytes that share a strong mutualistic association with the host plant. As targeted genome editing becomes more advanced, it is possible to envision the development of synthetic sensing and effector signal transduction systems to facilitate the regulation of designer genetic circuits of dynamic metabolic pathways between crops and endophytes.

## Conclusions

The opportunities for exploiting plant–microbe interactions for bioenergy crop production are numerous and diverse. The delivery of large volumes of low-cost biomass to replace existing fossil-based production must become a reality in the coming decades if we are to avoid catastrophic climate change. To achieve this at the same time as feeding the growing population is a major challenge for plant science. Land unfit for food production must be brought into use and planted with low-input perennial crops capable of producing high-biomass yields annually. Energy crops inoculated with beneficial endophytes can also be employed to phytoremediate land for future food production. Production costs must be kept to a minimum, both in economic and in energetic terms to supply low-cost sustainable biomass to the biorefinary supply chain. Low-tech applications include coating seeds with microbial biofilms as a direct method for inoculating seedlings with beneficial bacteria to aid plant growth and development. However, there are extensive possibilities for manipulation of the rhizosphere environment, by programming both the plant root exudates, and the bacterial sensing and response mechanisms. It is worth noting that the majority of the interactions in this zone are currently uncharacterized, and so it will be important to monitor soil, rhizosphere and endophyte populations. This will be a challenge as rhizosphere interactions are complex and dynamic, influenced by both addition and loss of individuals within the system (Badri *et al.*, 2009). In addition, it will be important to ensure no opportunistic pathogens have been inadvertently stimulated. Outbreaks of food poisoning from field-grown lettuce and other fresh fruit and vegetables demonstrate the gravity of this scenario (Rosenblueth and Martínez-Romero, 2006; reviewed by Nithya *et al.*, 2014). However, such modifications might in practice have major benefits to the soil, even for the subsequent crop in a rotation, similar to the potential benefit of endophytes in crop rotation to suppress nematodes (Sturz and Kimpinski, 2004). Furthermore, perennial energy crops are unlikely to form part of crop rotations where a build-up of potential human pathogens could be problematic.

Synthetic biology is already a reality in the development of novel enzymes and microbes for fermentation of biomass to fuels and other products. In the future, both plants and their beneficial symbionts will be modified to enhance biomass production for a growing population in a changing climate. Major targets for optimizing beneficial plant–microbe interactions include QS, bacterial motility, biofilm formation and the plant–microbe signalling pathways, particularly those specific to obligate endophytes. Furthermore, systematic screening of plants growing in extreme environments promises to yield novel endophytes harbouring genes and pathways conferring abiotic stress tolerance, and potentially IST, for optimization and application in energy crops destined for growth on marginal soils. The opportunities afforded by synthetic approaches, in conjunction with the minimal endophyte LEANOME, should yield a new paradigm in sustainable agriculture, with energy crops leading the way.

## References

[b1] Abeles FB, Morgan PW, Saltveit PE (1992). Ethylene in Plant Biology.

[b2] Antunes MS, Morey KJ, Smith JJ, Albrecht KD, Bowen TA, Zdunek JK, Troupe JF, Cuneo MJ, Webb CT, Hellinga HW, Medford JI (2011). Programmable ligand detection system in plants through a synthetic signal transduction pathway. PLoS ONE.

[b3] Arencibia AD, Vinagre F, Estevez Y, Bernal A, Perez J, Cavalcanti J, Santana I, Hemerly AS (2006). *Gluconacetobacter diazotrophicus* elicits a sugarcane defense response against a pathogenic bacteria *Xanthomonas albilineans*. Plant Signal Behav.

[b4] Arora S, Patel PN, Vanza MJ, Rao GG (2014). Isolation and characterization of endophytic bacteria colonizing halophyte and other salt tolerant plant species from coastal Gujarat. Afr. J. Microbiol. Res.

[b5] Assmus B, Hutzler P, Kirchhof G, Amann R, Lawrence JR, Hartmann A (1995). In situ localization of *Azospirillum brasilense* in the rhizosphere of wheat using fluorescently labeled, rRNA-targeted oligonucleotide probes and scanning confocal laser microscopy. Appl. Environ. Microbiol.

[b6] Bacilio-Jiménez M, Aguilar-Flores S, Ventura-Zapata E, Pérez-Campos E, Bouquelet S, Zenteno E (2003). Chemical characterization of root exudates from rice (*Oryza sativa*) and their effects on the chemotactic response of endophytic bacteria. Plant Soil.

[b7] Badri DV, Weir TL, Van Der Lelie D, Vivanco JM (2009). Rhizosphere chemical dialogues: plant–microbe interactions. Curr. Opin. Biotechnol.

[b8] Bais HP, Walker TS, Schweizer HP, Vivanco JM (2002). Root specific elicitation and antimicrobial activity of rosmarinic acid in hairy root cultures of *Ocimum basilicum*. Plant Physiol. Biochem.

[b9] Bais HP, Fall R, Vivanco JM (2004). Biocontrol of *Bacillus subtilis* against infection of *Arabidopsis* roots by *Pseudomonas syringae* is facilitated by biofilm formation and surfactin production. Plant Physiol.

[b10] Baldani JI, Baldani VL (2005). History on the biological nitrogen fixation research in graminaceous plants: special emphasis on the Brazilian experience. An. Acad. Bras. Ciênc.

[b500] Baldani JI, Baldani V, Seldin L, Döbereiner J (1986). Characterization of Herbaspirillum seropedicae gen. nov., sp. nov., a root-associated nitrogen-fixing bacterium. International Journal of Systematic Bacteriology.

[b11] Baldani VLD, Baldani JI, Döbereiner J (1987). Inoculation of field-grown wheat (*Triticum aestivum*) with *Azospirillum* spp. in Brazil. Biol. Fertil. Soils.

[b12] Baldani VLD, Baldani JI, Olivares FL, Döbereiner J (1992). Identification and ecology of *Herbaspirillum seropedicae* and closely related *Pseudomonas rubrisubalbicans*. Symbiosis.

[b13] Baldani JI, Oliveira ALM, Guimarães SL, Baldani VLD, Reis FB, Silva LG, Reis VM, Teixeira KRS, Döbereiner J (2000). Biological nitrogen fixation (BNF) in non-leguminous plants: the role of endophytic diazotrophs. Curr. Plant Sci. Biotechnol. Agric.

[b14] Bashan Y (1998). Inoculants of plant growth-promoting bacteria for use in agriculture. Biotechnol. Adv.

[b15] Berg G, Eberl L, Hartmann A (2005). The rhizosphere as a reservoir for opportunistic human pathogenic bacteria. Environ. Microbiol.

[b16] Berman-Frank I, Lundgren P, Falkowski P (2003). Nitrogen fixation and photosynthetic oxygen evolution in *Cyanobacteria*. Res. Microbiol.

[b17] Bertalan M, Albano R, de Pádua V, Rouws L, Rojas C, Hemerly A, Teixeira K, Schwab S, Araujo J, Oliveira A, França L, Magalhães V, Alquéres S, Cardoso A, Almeida W, Loureiro MM, Nogueira E, Cidade D, Oliveira D, Simão T, Macedo J, Valadão A, Dreschsel M, Freitas F, Vidal M, Guedes H, Rodrigues E, Meneses C, Brioso P, Pozzer L, Figueiredo D, Montano H, Junior J, de Souza Filho G, Martin QFV, Ferreira B, Branco A, Gonzalez P, Guillobel H, Lemos M, Seibel L, Macedo J, Alves-Ferreira M, Sachetto-Martins G, Coelho A, Santos E, Amaral G, Neves A, Pacheco AB, Carvalho D, Lery L, Bisch P, Rössle SC, Urményi T, Rael PA, Silva R, Rondinelli E, von Krüger W, Martins O, Baldani JI, Ferreira PCG (2009). Complete genome sequence of the sugarcane nitrogen-fixing endophyte *Gluconacetobacter diazotrophicus* Pal5. BMC Genomics.

[b18] Boddey RM, Urquiaga S, Reis VM, Döbereiner J (1991). Biological nitrogen fixation associated with sugar cane. Plant Soil.

[b19] Boddey RM, Oliveira OC, Urquiaga S, Reis VM, de Olivares FL, Baldani VLD, Döbereiner J (1995). Biological nitrogen fixation associated with sugar cane and rice: contributions and prospects for improvement. Plant Soil.

[b20] Bonfante P, Anca IA (2009). Plants, mycorrhizal fungi, and bacteria: a network of interactions. Annu. Rev. Microbiol.

[b200] Bottini R, Cassán F, Piccoli P (2004). Gibberellin production by bacteria and its involvement in plant growth promotion and yield increase. Appl. Microbiol. Biotechnol.

[b22] Buanafina MMDO, Langdon T, Hauck B, Dalton S, Morris P (2008). Expression of a fungal ferulic acid esterase increases cell wall digestibility of tall fescue (*Festuca arundinacea*. Plant Biotechnol. J.

[b23] Buanafina MMDO, Langdon T, Hauck B, Dalton S, Timms-Taravella E, Morris P (2010). Targeting expression of a fungal ferulic acid esterase to the apoplast, endoplasmic reticulum or golgi can disrupt feruloylation of the growing cell wall and increase the biodegradability of tall fescue (*Festuca arundinacea*. Plant Biotechnol. J.

[b24] Budiharjo A, Chowdhury SP, Dietel K, Beator B, Dolgova O, Fan B, Bleiss W, Ziegler J, Schmid M, Hartmann A, Borriss R (2014). Transposon mutagenesis of the plant-associated *Bacillus amyloliquefaciens* ssp. *plantarum* FZB42 revealed that the nfrA and RBAM17410 genes are involved in plant–microbe-interactions. PLoS ONE.

[b25] Bulgarelli D, Rott M, Schlaeppi K, van Themaat EVL, Ahmadinejad N, Assenza F, Rauf P, Huettel B, Reinhardt R, Schmelzer E, Peplies J, Gloeckner FO, Amann R, Eickhorst T, Schulze-Lefert P (2012). Revealing structure and assembly cues for Arabidopsis root-inhabiting bacterial microbiota. Nature.

[b27] Cai T, Cai W, Zhang J, Zheng H, Tsou AM, Xiao L, Zhong Z, Zhu J (2009). Host legume-exuded antimetabolites optimize the symbiotic rhizosphere. Mol. Microbiol.

[b28] Cao Q, Op den Camp R, Seifi Kalhor M, Bisseling T, Geurts R (2012). Efficiency of *Agrobacterium rhizogenes*–mediated root transformation of Parasponia and Trema is temperature dependent. Plant Growth Regul.

[b30] Compant S, Clément C, Sessitsch A (2010). Plant growth-promoting bacteria in the rhizo- and endosphere of plants: their role, colonization, mechanisms involved and prospects for utilization. Soil Biol. Biochem.

[b31] Conn VM, Walker AR, Franco CMM (2008). Endophytic *Actinobacteria* induce defense pathways in *Arabidopsis thaliana*. Mol. Plant Microbe Interact.

[b32] Dames JF, Ridsdale CJ (2012). What we know about arbuscular mycorhizal fungi and associated soil bacteria. Afr. J. Biotechnol.

[b33] Davis SC, Parton WJ, Dohleman FG, Smith CM, Grosso SD, Kent AD, DeLucia EH (2010). Comparative biogeochemical cycles of bioenergy crops reveal nitrogen-fixation and low greenhouse gas emissions in a *Miscanthus* × *giganteus* agro-ecosystem. Ecosystems.

[b34] Dennis PG, Miller AJ, Hirsch PR (2010). Are root exudates more important than other sources of rhizodeposits in structuring rhizosphere bacterial communities?. FEMS Microbiol. Ecol.

[b36] Döbereiner J, Reis VM, Paula MA, Olivares F (1993). Endophytic diazotrophs in sugar cane, cereals and tuber plants. Curr. Plant Sci. Biotechnol. Agric.

[b37] Dodds PN, Rathjen JP (2010). Plant immunity: towards an integrated view of plant–pathogen interactions. Nat. Rev. Genet.

[b38] Dong Z, Canny MJ, McCully ME, Roboredo MR, Cabadilla CF, Ortega E, Rodes R (1994). A nitrogen-fixing endophyte of sugarcane stems (a new role for the apoplast). Plant Physiol.

[b39] Doty SL, Oakley B, Xin G, Kang JW, Singleton G, Khan Z, Vajzovic A, Staley JT (2009). Diazotrophic endophytes of native black cottonwood and willow. Symbiosis.

[b40] Durbak A, Yao H, McSteen P (2012). Hormone signaling in plant development. Curr. Opin. Plant Biol.

[b41] Eckert B, Weber OB, Kirchhof G, Halbritter A, Stoffels M, Hartmann A (2001). *Azospirillum doebereinerae* sp. nov., a nitrogen-fixing bacterium associated with the C4-grass Miscanthus. Int. J. Syst. Evol. Microbiol.

[b42] Egorshina AA, Khairullin RM, Sakhabutdinova AR, Luk'yantsev MA (2011). Involvement of phytohormones in the development of interaction between wheat seedlings and endophytic *Bacillus subtilis* strain 11BM. Russ. J. Plant Physiol.

[b43] Elbeltagy A, Nishioka K, Sato T, Suzuki H, Ye B, Hamada T, Isawa T, Mitsui H, Minamisawa K (2001). Endophytic colonization and *in planta* nitrogen fixation by a *Herbaspirillum* sp. isolated from wild rice species. Appl. Environ. Microbiol.

[b45] Evangelisti E, Rey T, Schornack S (2014). Cross-interference of plant development and plant–microbe interactions. Curr. Opin. Plant Biol.

[b46] Feng Z, Zhang B, Ding W, Liu X, Yang DL, Wei P, Cao F, Zhu S, Zhang F, Mao Y, Zhu JK (2013). Efficient genome editing in plants using a CRISPR/Cas system.. Cell Res.

[b47] Finlay RD (2008). Ecological aspects of mycorrhizal symbiosis: with special emphasis on the functional diversity of interactions involving the extraradical mycelium. J. Exp. Bot.

[b48] Foster KR, Bell T (2012). Competition, not cooperation, dominates interactions among culturable microbial species. Curr. Biol.

[b49] Fowler M, Beck K (1999). Refactoring: Improving the Design of Existing Code.

[b50] Fuentes-Ramirez LE, Caballero-Mellado J, Sepúlveda J, Martínez-Romero E (1999). Colonization of sugarcane by *Acetobacter diazotrophicus* is inhibited by high N-fertilization. FEMS Microbiol. Ecol.

[b51] Gan HY, Gan HM, Savka MA, Triassi AJ, Wheatley MS, Smart LB, Fabio ES, Hudson AO (2014). Whole-genome sequences of 13 endophytic bacteria isolated from shrub willow (salix) grown in Geneva, New York]. Genome Announc.

[b52] Gillis M, Kersters K, Hoste B, Janssens D, Kroppenstedt RM, Stephan MP, Teixeira KRS, Dobereiner J, De Ley J (1989). *Acetobacter diazotrophicus* sp. nov., a nitrogen-fixing acetic acid bacterium associated with sugarcane. Int. J. Syst. Bacteriol.

[b53] Glick BR (2014). Bacteria with ACC deaminase can promote plant growth and help to feed the world. Microbiol. Res.

[b54] Goldfarb KC, Karaoz U, Hanson CA, Santee CA, Bradford MA, Treseder KK, Wallenstein MD, Brodie EL (2011). Differential growth responses of soil bacterial taxa to carbon substrates of varying chemical recalcitrance. Front. Microbiol.

[b55] Greenberg EP (1997). Quorum sensing in Gram-negative bacteria. ASM News.

[b56] Großkopf T, Soyer OS (2014). Synthetic microbial communities. Curr. Opin. Microbiol.

[b58] Hardoim PR, van Overbeek LS, Elsas JDV (2008). Properties of bacterial endophytes and their proposed role in plant growth. Trends Microbiol.

[b59] Heaton EA, Flavell RB, Mascia PN, Thomas SR, Dohleman FG, Long SP (2008). Herbaceous energy crop development: recent progress and future prospects. Curr. Opin. Biotechnol.

[b60] Hildebrandt U, Janetta K, Bothe H (2002). Towards growth of arbuscular mycorrhizal fungi independent of a plant host. Appl. Environ. Microbiol.

[b61] Hirsch AM (2004). Plant–microbe symbioses: a continuum from commensalism to parasitism. Symbiosis.

[b62] Hottes AK, Freddolino PL, Khare A, Donnell ZN, Liu JC, Tavazoie S (2013). Bacterial adaptation through loss of function. PLoS Genet.

[b63] Hungria M, Campo RJ, Souza EM, Pedrosa FO (2010). Inoculation with selected strains of *Azospirillum brasilense* and *A. lipoferum* improves yields of maize and wheat in Brazil. Plant Soil.

[b64] Hurek T, Reinhold-Hurek B, Van Montagu M, Kellenberger E (1994). Root colonization and systemic spreading of *Azoarcus* sp. strain BH72 in grasses. J. Bacteriol.

[b65] Iqbal A, Hasnain S (2013). Auxin producing *Pseudomonas* strains: biological candidates to modulate the growth of *Triticum aestivum* beneficially. Am. J. Plant Sci.

[b66] Isawa T, Yasuda M, Awazaki H, Minamisawa K, Shinozaki S, Nakashita H (2010). *Azospirillum* sp. strain B510 enhances rice growth and yield. Microbes Environ.

[b67] James EK (2000). Nitrogen fixation in endophytic and associative symbiosis. Field Crops Res.

[b68] James EK, Olivares FL (1998). Infection and colonization of sugar cane and other graminaceous plants by endophytic diazotrophs. Crit. Rev. Plant Sci.

[b69] James EK, Reis VM, Olivares FL, Baldani JI, Döbereiner J (1994). Infection of sugar cane by the nitrogen-fixing bacterium *Acetobacter diazotrophicus*. J. Exp. Bot.

[b70] James EK, Olivares FL, Baldani JI, Döbereiner J (1997). *Herbaspirillum*, an endophytic diazotroph colonizing vascular tissue in leaves of *Sorghum bicolor* L. Moench. J. Exp. Bot.

[b700] Jarboe LR, Zhang X, Wang X, Moore JC, Shanmugam KT, Ingram LO (2010). Metabolic engineering for production of biorenewable fuels and chemicals: contributions of synthetic biology. BioMed Research International.

[b71] Jones DL, Nguyen C, Finlay RD (2009). Carbon flow in the rhizosphere: carbon trading at the soil–root interface. Plant Soil.

[b72] Jones MB, Finnan J, Hodkinson TR (2014). Morphological and physiological traits for higher biomass production in perennial rhizomatous grasses grown on marginal land. Glob. Change Biol. Bioenergy.

[b73] Kachroo A, Robin GP (2013). Systemic signaling during plant defense. Curr. Opin. Plant Biol.

[b74] Keymer DP, Kent AD (2013). Contribution of nitrogen fixation to first year *Miscanthus* × *giganteus*. Glob. Change Biol. Bioenergy.

[b75] Khan Z, Doty SL (2009). Characterization of bacterial endophytes of sweet potato plants. Plant Soil.

[b76] Kim S, Lowman S, Hou G, Nowak J, Flinn B, Mei C (2012). Growth promotion and colonization of switchgrass (*Panicum virgatum*) cv. Alamo by bacterial endophyte *Burkholderia phytofirmans* strain PsJN. Biotechnol. Biofuels.

[b77] Kirchhof G, Reis VM, Baldani JI, Eckert B, Döbereiner J, Hartmann A, Malik KA, Ladha JK, de Bruijn FJ (1997). Occurrence, physiological and molecular analysis of endophytic diazotrophic bacteria in gramineous energy plants. Opportunities for Biological Nitrogen Fixation in Rice and Other Non-Legumes.

[b78] Kirchhof G, Baldani JI, Reis VM, Hartmann A (1998). Molecular assay to identify *Acetobacter diazotrophicus* and detect its occurrence in plant tissues. Can. J. Microbiol.

[b79] Kirchhof G, Eckert B, Stoffels M, Baldani JI, Reis VM, Hartmann A (2001). *Herbaspirillum frisingense* sp. nov., a new nitrogen-fixing bacterial species that occurs in C4-fibre plants. Int. J. Syst. Evol. Microbiol.

[b80] Kloepper JW, Ryu C-M (2006). Bacterial endophytes as elicitors of induced systemic resistance. Soil Biol.

[b81] Kusari P, Kusari S, Lamshöft M, Sezgin S, Spiteller M, Kayser O (2014). Quorum quenching is an antivirulence strategy employed by endophytic bacteria. Appl. Microbiol. Biotechnol.

[b82] Lakshmanan V, Kitto SL, Caplan JL, Hsueh Y-H, Kearns DB, Wu Y-S, Bais HP (2012). Microbe-associated molecular patterns-triggered root responses mediate beneficial rhizobacterial recruitment in *Arabidopsis*. Plant Physiol.

[b84] Leite MCBS, de Farias ARB, Freire FJ, Andreote FD, Sobral JK, Freire MBGS (2014). Isolation, bioprospecting and diversity of salt-tolerant bacteria associated with sugarcane in soils of Pernambuco, Brazil. Rev. Bras. Eng. Agríc. Amb.

[b85] van der Lelie D, Taghavi S, Monchy S, Schwender J, Miller L, Ferrieri R, Rogers A, Wu X, Zhu W, Weyens N, Vangronsveld J, Newman L (2009). Poplar and its bacterial endophytes: coexistence and harmony. Crit. Rev. Plant Sci.

[b86] Lery LM, Hemerly AS, Nogueira EM, von Krüger WM, Bisch PM (2011). Quantitative proteomic analysis of the interaction between the endophytic plant-growth-promoting bacterium *Gluconacetobacter diazotrophicus* and sugarcane. Mol. Plant Microbe Interact.

[b87] Lewandowski I, Scurlock JM, Lindvall E, Christou M (2003). The development and current status of perennial rhizomatous grasses as energy crops in the US and Europe. Biomass Bioenergy.

[b89] Looger LL, Dwyer MA, Smith JJ, Hellinga HW (2003). Computational design of receptor and sensor proteins with novel functions. Nature.

[b90] van Loon LC, Bakker PA, Pieterse CM (1998). Systemic resistance induced by rhizosphere bacteria. Annu. Rev. Phytopathol.

[b91] Lundberg DS, Lebeis SL, Paredes SH, Yourstone S, Gehring J, Malfatti S (2012). Defining the core, *Arabidopsis thaliana* root microbiome. Nature.

[b92] Luo S, Xu T, Chen L, Chen J, Rao C, Xiao X, Wan Y, Zeng G, Long F, Liu C, Liu Y (2012). Endophyte-assisted promotion of biomass production and metal-uptake of energy crop sweet sorghum by plant-growth-promoting endophyte *Bacillus* sp. SLS18. Appl. Microbiol. Biotechnol.

[b93] Magnani GS, Didonet CM, Cruz LM, Picheth CF, Pedrosa FO, Souza EM (2010). Diversity of endophytic bacteria in Brazilian sugarcane. Genet. Mol. Res.

[b94] Malik KA, Bilal R, Mehnaz S, Rasul G, Mirza MS, Ali S (1997). Association of nitrogen-fixing, plant-growth-promoting rhizobacteria (PGPR) with kallar grass and rice. Plant Soil.

[b95] Mark GL, Dow JM, Kiely PD, Higgins H, Haynes J, Baysse C, Abbas A, Foley T, Franks A, Morrissey J, O'Gara F (2005). Transcriptome profiling of bacterial responses to root exudates identifies genes involved in microbe–plant interactions. Proc. Natl Acad. Sci. USA.

[b96] Mastretta C, Taghavi S, Van Der Lelie D, Mengoni A, Galardi F, Gonnelli C, Barac T, Boulet J, Weyens N, Vangronsveld J (2009). Endophytic bacteria from seeds of *Nicotiana tabacum* can reduce cadmium phytotoxicity. Int. J. Phytorem.

[b97] Mathesius U (2009). Comparative proteomic studies of root–microbe interactions. J. Proteom.

[b98] Mei C, Flinn BS (2010). The use of beneficial microbial endophytes for plant biomass and stress tolerance improvement. Recent Pat. Biotechnol.

[b99] Mendes R, Pizzirani-Kleiner AA, Welington LA, Raaijmakers JM (2007). Diversity of cultivated endophytic bacteria from sugarcane: genetic and biochemical characterization of *Burkholderia cepacia* complex isolates. Appl. Environ. Microbiol.

[b100] Mitter B, Petric A, Shin MW, Chain PS, Hauberg-Lotte L, Reinhold-Hurek B, Nowak J, Sessitsch A (2013). Comparative genome analysis of *Burkholderia phytofirmans* PsJN reveals a wide spectrum of endophytic lifestyles based on interaction strategies with host plants. Front. Plant Sci.

[b101] Miyamoto T, Kawahara M, Minamisawa K (2004). Novel endophytic nitrogen-fixing clostridia from the grass *Miscanthus sinensis* as revealed by terminal restriction fragment length polymorphism analysis. Appl. Environ. Microbiol.

[b102] Monteiro RA, Balsanelli E, Tuleski T, Faoro H, Cruz LM, Wassem R, de Baura VA, Tadra-Sfeir MZ, Weiss V, DaRocha WD, Muller-Santos M, Chubatsu LS, Huergo LF, Pedrosa FO, de Souza EM (2012a). Genomic comparison of the endophyte *Herbaspirillum seropedicae* SmR1 and the phytopathogen *Herbaspirillum rubrisubalbicans* M1 by suppressive subtractive hybridization and partial genome sequencing. FEMS Microbiol. Ecol.

[b103] Monteiro RA, Alsanelli E, Wassem R, Marin AM, Brusamarello-Santos LCC, Schmidt MA, Tadra-Sfeir MZ, Pankievicz VCS, Cruz LM, Chubatsu LS, Pedrosa FO, Souza EM (2012b). *Herbaspirillum*–plant interactions: microscopical, histological and molecular aspects. Plant Soil.

[b105] Moran NA, McLaughlin HJ, Sorek R (2009). The dynamics and time scale of ongoing genomic erosion in symbiotic bacteria. Science.

[b106] Naveed M, Hussain B, Zahir A, Mitter B, Sessitsch A (2014a). Drought stress amelioration in wheat through inoculation with *Burkholderia phytofirmans* strain PsJN. Plant Growth Regul.

[b107] Naveed M, Mitter B, Reichenauer TG, Wieczorek K, Sessitsch A (2014b). Increased drought stress resilience of maize through endophytic colonization by *Burkholderia phytofirmans* PsJN and Enterobacter sp. FD17. Environ. Exp. Bot.

[b108] Newman MA, Sundelin T, Nielsen JT, Erbs G (2013). MAMP (microbe-associated molecular pattern) triggered immunity in plants. Front. Plant Sci.

[b109] Nithya A, Gothandam KM, Babu S (2014). Alternative ecology of human pathogenic bacteria in fruits and vegetables. Plant Pathol. J.

[b110] Okon Y, Itzigsohn R (1995). The development of *Azospirillum* as a commercial inoculant for improving crop yields. Biotechnol. Adv.

[b111] Oldroyd EDG, Murray JD, Poole PS, Downie JA (2011). The rules of engagement in the legume-rhizobial symbiosis. Annu. Rev. Genet.

[b112] Olivares FL, Baldani VLD, Reis VM, Baldani JI, Döbereiner J (1996). Occurrence of the endophytic diazotrophs *Herbaspirillum* spp. in roots, stems, and leaves, predominantly of Gramineae. Biol. Fertil. Soils.

[b113] de Oliveira ZM, Floh EI, Ferrara FI, Barbosa HR (2011). Diazotrophyc rhizobacteria isolated from sugarcane can release amino acids in a synthetic culture medium. Biol. Fertil. Soils.

[b114] Onofre-Lemus J, Hernández-Lucas I, Girard L, Caballero-Mellado J (2009). ACC (1-aminocyclopropane-1-carboxylate) deaminase activity, a widespread trait in *Burkholderia* species, and its growth-promoting effect on tomato plants. Appl. Environ. Microbiol.

[b115] Pariona-Llanos R, Ibañez de Santi Ferrara F, Soto Gonzales HH, Barbosa HR (2010). Influence of organic fertilization on the number of culturable diazotrophic endophytic bacteria isolated from sugarcane. Eur. J. Soil Biol.

[b116] Paungfoo-Lonhienne C, Lonhienne TG, Yeoh A, Yun K, Webb IR, Lakshmanan P, Chan C, Xin L, Phaik-Eem RMA, Schmidt S, Hugenholtz P (2014). A new species of Burkholderia isolated from sugarcane roots promotes plant growth. Microb. Biotechnol.

[b117] Pedrosa FO (2011). Genome of *Herbaspirillum seropedicae* strain SmR1, a specialized diazotrophic endophyte of tropical grasses. PLoS Genet.

[b118] Pereira JA, Baldani JI (1995). Selection of *Azospirillum* spp. and *Herbaspirillum seropedicae* strains to inoculate rice and maize plants. In International Symposium on Sustainable Agriculture for the Tropics—the role of Biological Nitrogen Fixation.

[b119] Pieterse CMJ (1998). A novel signaling pathway controlling induced systemic resistance in Arabidopsis. Plant Cell.

[b121] Puchta H, Fauser F (2014). Synthetic nucleases for genome engineering in plants: prospects for a bright future. Plant J.

[b122] Qin S, Xing K, Jiang JH, Xu LH, Li WJ (2011). Biodiversity, bioactive natural products and biotechnological potential of plant-associated endophytic actinobacteria. Appl. Microbiol. Biotechnol.

[b124] Rajesh PS, Ravishankar RV (2013). Quorum quenching activity in cell-free lysate of endophytic bacteria isolated from *Pterocarpus santalinus* Linn., and its effect on quorum sensing regulated biofilm in *Pseudomonas aeruginosa* PAO1. Microbiol. Res.

[b126] Ramey BE, Koutsoudis M, von Bodman SB, Fuqua C (2004). Biofilm formation in plant–microbe associations. Curr. Opin. Microbiol.

[b127] Ratón TDLMO, Yano R, Gámez OR, Floh EIS, Díaz MDJS, Barbosa HR (2012). Isolation and characterisation of aerobic endospore forming Bacilli from sugarcane rhizosphere for the selection of strains with agriculture potentialities. World J. Microbiol. Biotechnol.

[b128] Reinhold-Hurek B, Hurek T (2011). Living inside plants: bacterial endophytes. Curr. Opin. Plant Biol.

[b129] Rogers A, McDonald K, Muehlbauer MF, Hoffman A, Koenig K, Newman L, Taghavi S, Lelie D (2012). Inoculation of hybrid poplar with the endophytic bacterium Enterobacter sp. 638 increases biomass but does not impact leaf level physiology. GCB Bioenergy.

[b130] Rosenblueth M, Martínez-Romero E (2006). Bacterial endophytes and their interactions with hosts. Mol. Plant Microbe Interact.

[b132] Rothballer M, Eckert B, Schmid M, Fekete A, Schloter M, Lehner A, Pollmann S, Hartmann A (2008). Endophytic root colonization of gramineous plants by *Herbaspirillum frisingense*. FEMS Microbiol. Ecol.

[b133] Rouws LF, Simões-Araújo JL, Hemerly AS, Baldani JI (2008). Validation of a Tn5 transposon mutagenesis system for *Gluconacetobacter diazotrophicus* through characterization of a flagellar mutant. Arch. Microbiol.

[b134] Ryan RP, Germaine K, Franks A, Ryan DJ, Dowling DN (2008). Bacterial endophytes: recent developments and applications. FEMS Microbiol. Lett.

[b135] Santi C, Bogusz D, Franche C (2013). Biological nitrogen fixation in non-legume plants. Ann. Bot.

[b137] Serrano L (2007). Synthetic biology: promises and challenges. Mol. Syst. Biol.

[b139] Sessitsch A, Hardoim P, Döring J, Weilharter A, Krause A, Woyke T, Mitter B, Hauberg-Lotte L, Friedrich F, Rahalkar M, Hurek T, Sarkar A, Bodrossy L, van Overbeek L, Brar D, van Elsas JD, Reinhold-Hurek B (2012). Functional characteristics of an endophyte community colonizing rice roots as revealed by metagenomic analysis. Mol. Plant Microbe Interact.

[b140] Shidore T, Dinse T, Öhrlein J, Becker A, Reinhold-Hurek B (2012). Transcriptomic analysis of responses to exudates reveal genes required for rhizosphere competence of the endophyte *Azoarcus* sp. strain BH72. Environ. Microbiol.

[b141] Simmons BA, Loque D, Blanch HW (2008). Next-generation biomass feedstocks for biofuel production. Genome Biol.

[b143] Smith SE, Smith FA (2011). Roles of arbuscular mycorrhizas in plant nutrition and growth: new paradigms from cellular to ecosystem scales. Annu. Rev. Plant Biol.

[b204] Spaepen S, Vanderleyden J, Remans R (2007). Indole-3-acetic acid in microbial and microorganism-plant signaling. FEMS Microbiol. Rev.

[b144] Steenhoudt O, Vanderleyden J (2000). *Azospirillum,* a free-living nitrogen-fixing bacterium closely associated with grasses: genetic, biochemical and ecological aspects. FEMS Microbiol. Rev.

[b145] Stewart EJ (2012). Growing unculturable bacteria. J. Bacteriol.

[b146] Straub D, Rothballer M, Hartmann A, Ludewig U (2013a). The genome of the endophytic bacterium *H. frisingense* GSF30(T) identifies diverse strategies in the *Herbaspirillum* genus to interact with plants. Front. Microbiol.

[b147] Straub D, Yang H, Liu Y, Tsap T, Ludewig U (2013b). Root ethylene signalling is involved in *Miscanthus sinensis* growth promotion by the bacterial endophyte *Herbaspirillum frisingense* GSF30T. J. Exp. Bot.

[b148] Sturz AV, Kimpinski J (2004). Endoroot bacteria derived from marigolds (*Tagetes* spp.) can decrease soil population densities of root-lesion nematodes in the potato root zone. Plant Soil.

[b150] Sun N, Zhao H (2013). Transcription activator-like effector nucleases (TALENs): a highly efficient and versatile tool for genome editing. Biotechnol. Bioeng.

[b151] Suto M, Takebayashi M, Saito K, Tanaka M, Yokota A, Tomita F (2002). Endophytes as producers of xylanase [online]. J. Biosci. Bioeng.

[b152] Taghavi S, Garafola C, Monchy S, Newman L, Hoffman A, Weyens N, Barac T, Vangronsveld J, Daniel van der Lelie D (2009). Genome survey and characterization of endophytic bacteria exhibiting a beneficial effect on growth and development of poplar trees. Appl. Environ. Microbiol.

[b153] Taghavi S, Garafola C, Monchy S, Newman L, Hoffman A, Weyens N, Barac T, Vangronsveld J, van der Lelie D (2010). Genome sequence of the plant growth promoting endophytic bacterium *Enterobacter* sp. 638. PLoS Genet.

[b154] Temme K, Dehua Z, Voigt CA (2012). Refactoring the nitrogen fixation gene cluster from *Klebsiella oxytoca*. Proc. Natl Acad. Sci. USA.

[b155] Theocharis A, Bordiec S, Fernandez O, Paquis S, Dhondt-Cordelier S, Baillieul F, Clément C, Barka EA (2012). *Burkholderia phytofirmans* PsJN primes *Vitis vinifera* L. and confers a better tolerance to low nonfreezing temperatures. Mol. Plant Microbe Interact.

[b156] Tikhonovich IA, Provorov NA (2011). Microbiology is the basis of sustainable agriculture: an opinion. Ann. Appl. Biol.

[b157] Tilman D (1998). The greening of the green revolution. Nature.

[b158] Toft C, Andersson SVG (2010). Evolutionary microbial genomics: insights into bacterial host adaptation. Nat. Rev. Genet.

[b205] Tsavkelova EA, Klimova SY, Cherdyntseva TA, Netrusov AI (2006). Hormones and hormone-like substances of microorganisms: A review. Appl. Biochem. Microbiol.

[b159] Tuck G, Glendining MJ, Smith P, House JI, Wattenbach M (2006). The potential distribution of bioenergy crops in Europe under present and future climate. Biomass Bioenergy.

[b160] Turner TR, Ramakrishnan K, Walshaw J, Heavens D, Alston M, Swarbreck D, Osbourn A, Grant A, Poole PS (2013). Comparative metatranscriptomics reveals kingdom level changes in the rhizosphere microbiome of plants. ISME J.

[b161] Umenhoffer K, Fehér T, Balikó G, Ayaydin F, Pósfai J, Blattner FR, Pósfai G (2010). Reduced evolvability of *Escherichia coli* MDS42, an IS-less cellular chassis for molecular and synthetic biology applications. Microb. Cell Fact.

[b162] Van Aken B, Peres CM, Doty SL, Yoon JM, Schnoor JL (2004). *Methylobacterium populi* sp. nov., a novel aerobic, pink-pigmented, facultatively methylotrophic, methane-utilizing bacterium isolated from poplar trees (*Populus deltoids *×* nigra* DN34). Int. J. Syst. Evol. Microbiol.

[b164] Vartoukian SR, Palmer RM, Wade WG (2010). Strategies for culture of ‘unculturable'bacteria. FEMS Microbiol. Lett.

[b165] Velázquez-Hernández ML, Baizabal-Aguirre VM, Cruz-Vázquez F, Trejo-Contreras MJ, Fuentes-Ramírez LE, Bravo-Patiño A, Valdez-Alarcón JJ (2011). *Gluconacetobacter diazotrophicus* levansucrase is involved in tolerance to NaCl, sucrose and desiccation, and in biofilm formation. Arch. Microbiol.

[b166] Videira SS, Oliveira DM, Morais RF, Borges WL, Baldani VLD, Baldani JI (2012). Genetic diversity and plant growth promoting traits of diazotrophic bacteria isolated from two *Pennisetum purpureum* Schum. genotypes grown in the field. Plant Soil.

[b167] Walker TS, Bais HP, Grotewold E, Vivanco JM (2003). Root exudation and rhizosphere biology. Plant Physiol.

[b168] Werner JJ, Knights D, Garciac ML, Scalfone NB, Smith S, Yarasheski K, Cummings TA, Beerse AR, Knight R, Angenenta LT (2011). Bacterial community structures are unique and resilient in full-scale bioenergy systems. Proc. Natl Acad. Sci. USA.

[b169] Weyens N, van der Lelie D, Taghavi S, Newman L, Vangronsveld J (2009a). Exploiting plant–microbe partnerships to improve biomass production and remediation. Trends Biotechnol.

[b170] Weyens N, van der Lelie D, Taghavi S, Vangronsveld J (2009b). Phytoremediation: plant–endophyte partnerships take the challenge. Curr. Opin. Biotechnol.

[b171] Wirthmueller L, Maqbool A, Banfield MJ (2013). On the front line: structural insights into plant–pathogen interactions. Nat. Rev. Microbiol.

[b172] Yang J, Kloepper JW, Ryu CM (2009). Rhizosphere bacteria help plants tolerate abiotic stress. Trends Plant Sci.

[b173] Ye B, Saito A, Minamisawa K (2005). Effect of inoculation with anaerobic nitrogen-fixing consortium on salt tolerance of *Miscanthus sinensis*. Soil Sci. Plant Nutr.

[b206] Yuan Z-C, Haudecoeur E, Faure D, Kerr KF, Nester EW (2008). Comparative transcriptome analysis of *Agrobacterium tumefaciens* in response to plant signal salicylic acid, indole-3-acetic acid and gamma-amino butyric acid reveals signalling cross-talk and Agrobacterium–plant co-evolution. Cell. Microbiol.

[b175] Zhang N, Wang D, Liu Y, Li S, Shen Q, Zhang R (2014). Effects of different plant root exudates and their organic acid components on chemotaxis, biofilm formation and colonization by beneficial rhizosphere-associated bacterial strains. Plant Soil.

[b176] Zhao L (2010). Genomics: the tale of our other genome. Nature.

[b207] Zinniel DK, Lambrecht P, Harris NB, Feng Z, Kuczmarski D, Higley P, Vidaver AK (2002). Isolation and characterization of endophytic colonizing bacteria from agronomic crops and prairie plants. Appl. Environ. Microbiol.

[b177] Zuccaro A, Lahrmann U, Güldener U, Langen G, Pfiffi S, Biedenkopf D, Wong W, Samans B, Grimm C, Basiewicz M, Murat C, Martin F, Kogel KH (2011). Endophytic life strategies decoded by genome and transcriptome analyses of the mutualistic root symbiont *Piriformospora indica*. PLoS Pathog.

